# Volatile organic compound profiles in oat are shaped by arbuscular mycorrhizal fungi, barley yellow dwarf virus and soil herbicide residues

**DOI:** 10.1080/15592324.2026.2737115

**Published:** 2026-09-23

**Authors:** Benjamin Fuchs, Lena Falk Nielsen, Valter Weijola, James D. Blande

**Affiliations:** a Department of Agroecology, Aarhus University, Slagelse, Denmark; b Department of Biodiversity Sciences, University of Turku, Turku, Finland; c Department of Environmental and Biological Sciences, University of Eastern Finland, Kuopio, Finland

**Keywords:** Induced plant defenses, stress physiology, chemical phenotyping, plant chemical ecology, plant soil interactions

## Abstract

Volatile organic compounds (VOCs) are emitted by plants and vary in quantity and composition depending on biotic and abiotic environmental conditions. Plant-emitted VOCs are altered by attacking herbivores and pathogens, and have been increasingly found to be fine-tuned by microbial associates and environmental pollutants. Here, we studied whether arbuscular mycorrhizal fungi (AMF) treatment, barley yellow dwarf virus (BYDV) infection, and soil pollution with glyphosate-based herbicide (GBH) residues affect the emission of volatile organic compounds by oat (*Avena sativa*) plants. Oat plants were grown in a common garden study in southwestern Finland, where seeds in half of the plots had received a dressing with AMF inoculant before being sown in plots with a history of regular glyphosate application and control plots without a history of glyphosate use. During the study, an average of 30% of the oat plants showed symptoms of infection by BYDV, which was then included as a treatment. We collected plant VOCs of eight plants per treatment via headspace sampling with subsequent analysis by gas chromatography coupled to mass spectrometry (GC-MS). The percentage of BYDV-infected plants did not differ between AMF-treated and untreated plants, nor between plants growing in glyphosate-polluted and glyphosate-free soil. The emission of VOCs displayed characteristic profiles depending on the soil GBH history, BYDV infection, and AMF seed treatment. In particular, AMF treatment caused an increase in the terpenoid (*E*)-*β*-ocimene, and the fatty acid derivatives 1-octen-3-ol and octanal. Virus infection reduced the emission of octanal and induced the emission of the terpenoids (*E*)-caryophyllene and *β*-farnesene. GBH-treated soil caused an increase in the emission of green leaf volatiles (*Z*)-3-hexen-1-ol, 1-hexanol, and (*Z*)-3-hexenyl-acetate compared to plants in control soil. Plant-emitted VOCs displayed a distinct pattern elicited by viral infection, fungal-root association, and herbicide pollution in soil, indicating the impact of biotic and abiotic factors on the plant's biology. While the biological mechanisms behind the distinct VOC profiles remain speculative, the results indicate that VOC emission patterns may be utilized to infer plant and soil health status in crop production.

## Introduction

Biological systems respond to biotic and abiotic challenges with a multitude of fine-tuned adjustments.^1^ ^,^ ^2^ Challenging environmental stimuli, including microbial diseases, can cause severe performance and fitness effects in plants and animals, and are projected to increase in predicted climate change scenarios.^3^ ^,^ ^4^ Early detection of these diseases is essential for timely, targeted treatment to limit their spread and impact.^5^ ^,^ ^6^

Moreover, agricultural cropping systems must withstand a number of biotic and abiotic stressors, such as drought, UV, ozone, soil quality, and particularly a multitude of harmful organisms including pest insects and pathogens, which can greatly impact crop performance and ultimately reduce yield.^7^ ^,^ ^8^ Monocropping yield-selected annual cultivars is the most common form of crop production globally.^9^ The benefits of high-yield monocropping often correlates with a vulnerability to pest outbreaks, which in turn increases the reliance on synthetic pesticides.^10^ ^,^ ^11^ Ongoing climate change, extreme abiotic conditions, and the introduction of new pest species pose a major challenge to crop production, further increasing the dependence on agrochemical inputs, causing an increasing legacy of pollution in soil.^12-15^

Soil constitutes a complex matrix of biotic and abiotic factors that critically influence crop performance. Therefore, manipulation of soil parameters, particularly the microbial community, is being increasingly utilized to improve crop health.^16^ ^,^ ^17^ Assessing and improving soil conditions is one of the current challenges, as soil degradation has been identified as the leading detrimental factor responsible for yield reduction during the past decades.^18-20^ The exuberated use of pesticides contributes largely to the degradation of soil multifunctionality and ecosystem services in agriculture.^14^ ^,^ ^21-23^ Pesticide residues in soil affect plant biosynthetic pathways, including secondary metabolite production, which plays a critical role in mediating plant–insect interactions and supporting ecosystem services such as pollination and pest control.^24-29^

On the other hand, microbial plant symbionts in the rhizosphere often shape the aboveground metabolite concentrations in plants.^30^ Colonization by arbuscular mycorrhizal fungi (AMF) species such as *Glomus mosseae* and *Rhizophagus irregularis* has been shown to boost emission of defense-related volatile organic compounds (VOCs) like (*E*)-caryophyllene, *β*-farnesene, and methyl salicylate,^31^ ^,^ ^32^ which are signaling molecules for herbivores and their predators.^33^

Furthermore, VOCs are emerging as diagnostic indicators of plant health, often revealing pathogen infections before visible symptoms appear.^34^ ^,^ ^35^ Specific emission patterns, such as an increase in green leaf volatile (GLV) emissions, have been linked to early-stage infections like late blight in potatoes.^36^ Similar shifts in VOC patterns have been reported in wheat and barley infected by barley yellow dwarf virus (BYDV), suggesting a conserved plant response to BYDV at the volatile level.^37^ ^,^ ^38^

Timely countermeasures to viral and fungal infections and insect infestations require the detection and identification of a threat in the early stages, which in many instances relies on visible plant symptoms, such as deformation, coloration, and damage^39^ or in-depth laboratory analyzes.^6^ Precision agriculture is advancing the field of pest control by implementing high-precision spectral diagnosis tools which can identify visual plant cues as disease symptoms at the individual plant level.^40-42^ Unfortunately, by the time visual disease symptoms are evident, the damage to the crop is often advanced and incurs a reduction in yield together with high costs of often environmentally harmful countermeasures.^41^ ^,^ ^43^ Besides visual cues, plants emit VOCs as semiochemicals that provide information on the plant health status.^44^ ^,^ ^45^ Plant VOCs have been demonstrated to be altered by various biotic and abiotic factors and are a potentially promising indicator for the early diagnosis of pest problems at the individual plant and field scales.^46^

Here, we analyzed the VOC pattern of oat plants and hypothesize that glyphosate-based herbicide (GBH) treatment, seed dressing with a commercially available AMF inoculant, as well as infection by BYDV, alters the VOC emission in its qualitative composition.

## Methods

### Experimental design

The field experiment was carried out in 2024 at the University of Turku's Ruissalo Botanical Garden in southwestern Finland (60°26′N, 22°10′E). The site is characterized by medium-clay soil with a high organic matter content (>120 g kg⁻^1^).^47^ The experimental area comprised 24 plots (6 × 1.2 m), with adjacent plots separated by 1 m buffer zones. Twelve plots have received the glyphosate-based herbicide (GBH) Roundup Gold annually since 2014 at the rate of 6.4 L ha⁻^1^ (equivalent to 3 kg glyphosate active ingredient ha⁻^1^), while the remaining 12 plots have received an equivalent volume of water and served as controls (for details see^48^). Before the GBH application in spring, the average glyphosate concentration in the treated plots was 2.30 mg/kg, compared with 0.012 mg/kg in the control plots. During the experimental period (May to August), our experimental setup had three treatment groups: control, GBH, and AMF.

Plots were divided into two equally sized plots of 2.5 m length, where the identical plant setup was sown, either with or without mycorrhizal seed inoculation. Oat (*Avena sativa* L.) and faba bean (*Vicia faba* L.) were planted alternating in four rows over a length of two meters in each subplot with a row distance of 20 cm. The intercropping aspect is not part of this study and is only mentioned for completeness of the study description.

### Treatments

The AMF treatment was established via a dressing of mycorrhizal fungal spores before sowing. The commercially available inoculant included spores of *Rhizophagus irregularis*, *Funneliformis mosseae,* and *Septoglomus deserticola*, and a sticky agent (Mycovivo, symbiom.cz). All seeds were moistened with tap water, and approx. 5 kg of seeds were hand-mixed for 10 minutes with 50 grams of AMF inoculant according to the manufactures instructions to ensure homogenous coating of the seeds. The same treatment was applied to control seeds but with heat-sterilized AMF inoculant. AMF treatment was included as an experimental treatment to investigate its potential effects on plant VOC emissions, but we therefore cannot directly confirm the degree of root colonization.

During June, the first signs of barley yellow dwarf virus (BYDV) were recorded on the oat plants and monitored weekly until the end of the study in July. Infected oat plants showed pronounced chlorosis starting at the leaf tips and moving downward, with some leaves displaying reddish-purple margins. Symptoms occurred in irregular patches across the plots. Due to the high incidence of BYDV infection throughout the plots, we included BYDV as an ex-tempore treatment for later VOC analyzes.

During the mornings (10–12 a.m.) of three consecutive days in the middle of July, we collected VOCs from oat of eight replicates per treatment. Dates were selected according to weather forcast with days of similar weather conditions. We included the following four treatments for VOC collection: control, control + AMF, control + BYDV, and GBH. We did not collect VOCs from the treatment combinations GBH + AMF, or GBH + BYDV due to practical limitations.

The BYDV infection status of each plot was assessed weekly throughout the growing season by visually inspecting all plants. Infection severity was estimated based on the percentage of leaf area showing symptoms on the main shoot leaves of infected plants and evaluated using a 1–6 scale: 1 = 1%–10%, 2 = 10%–20%, 3 = 20%–30%, 4 = 30%–40%, 5 = 40%–50%, and 6 = > 50%.

### VOC collection

In total, we collected samples from four treatments via dynamic headspace sampling: (1) BYDV symptom-free plants in eight control plots, (2) BYDV-infected plants (damage category 3–5) from control plots, (3) BYDV symptom-free plants from plots treated with mycorrhizal seed inoculation, and (4) BYDV symptom-free plants from eight plots with a history of GBH application. During VOC collection, plants were shaded with a tent structure to avoid water accumulation from transpiration in the collection bags and VOCs were sampled for a total of one hour following published protocols.^49^ One replicate consisted of three plant individuals, enclosed in an airtight polyethylene terephthalate oven bag (25 × 38  cm; Look® Uunipussi Eskimo Oy). Plants were of similar size, and biomass was measured subsequent to VOC collection from dried plant material to quantify the emission rate per gram plant material. One opening in the bag was used for the introduction of purified air (220 ml/min) and on the opposite side of the bag we inserted a stainless-steel tube filled with 200 mg Tenax TA 60/80 adsorbent (Markes International Ltd), which trapped the VOCs and was connected to a vacuum pump (KNF) with a flow rate of 200 ml/min for 1 h.^26^ VOCs were collected from four bags (each treatment) simultaneously. We calibrated airflows with a flowmeter every morning before the trapping started using a bubble film calibrator (mini-Buck Calibrator, Buck). VOC samples were analyzed by gas chromatography–mass spectrometry (GC–MS), and ChemStation software was used for further compound identification and quantification.

### GCMS analysis

To minimize VOC loss, samples were stored at 4 °C until GC–MS analysis. VOCs were thermally desorbed at 250 °C for 10 minutes using a TD-100 unit (Markes International Ltd), cryofocused at −10 °C, and transferred in splitless mode onto an HP-5 capillary column (60 m × 250 μm × 0.25 μm; Agilent), following established protocol in those 2 publications.^26^ ^,^ ^50^ Compounds were identified by comparison with pure standards and the NIST 20 library. Quantification was based on external standards comprising 21 terpenoids and nine green leaf volatiles; compounds lacking authentic standards were quantified using chemically similar compounds (e.g. *α*-pinene for monoterpenes). VOC emissions were expressed as ng g⁻^1^ min⁻^1^ of dry leaf mass. Most detected compounds were green leaf volatiles, monoterpenes, or sesquiterpenes.

### Statistical analyzes

The incidence of virus infection across plots was evaluated separately for each week using Fisher's exact test. Differences in overall VOC profiles were explored using nonmetric multidimensional scaling (NMDS) based on all 28 detected compounds. Bray–Curtis dissimilarities were calculated with Wisconsin double standardization, and the data were ordinated in two dimensions using vegan:metaMDS (vegan:vegdist; stress = 0.167). The effects of soil GBH residues, BYDV infection, AMF treatment, and their interactions on VOC composition were assessed using PERMANOVA with 9999 permutations (vegan:adonis).

For individual VOCs, concentrations were compared among the four treatment groups using one-way ANOVA. Data were assessed for normality and homogeneity of variance using Shapiro–Wilk and Levene's tests, respectively, and log-transformed when required. Significant treatment effects were followed by Tukey's HSD post-hoc comparisons, with false discovery rate correction applied to account for multiple testing. Random Forest analysis was additionally used to determine which VOCs were most effective at distinguishing among treatments. Compounds were ranked according to mean decrease in accuracy, and standardized VOC abundances (z-scores) were visualized using a heatmap. All statistical analyzes were performed in R (v. 4.5.1).

## Results

### Virus infection

**Figure 1.**
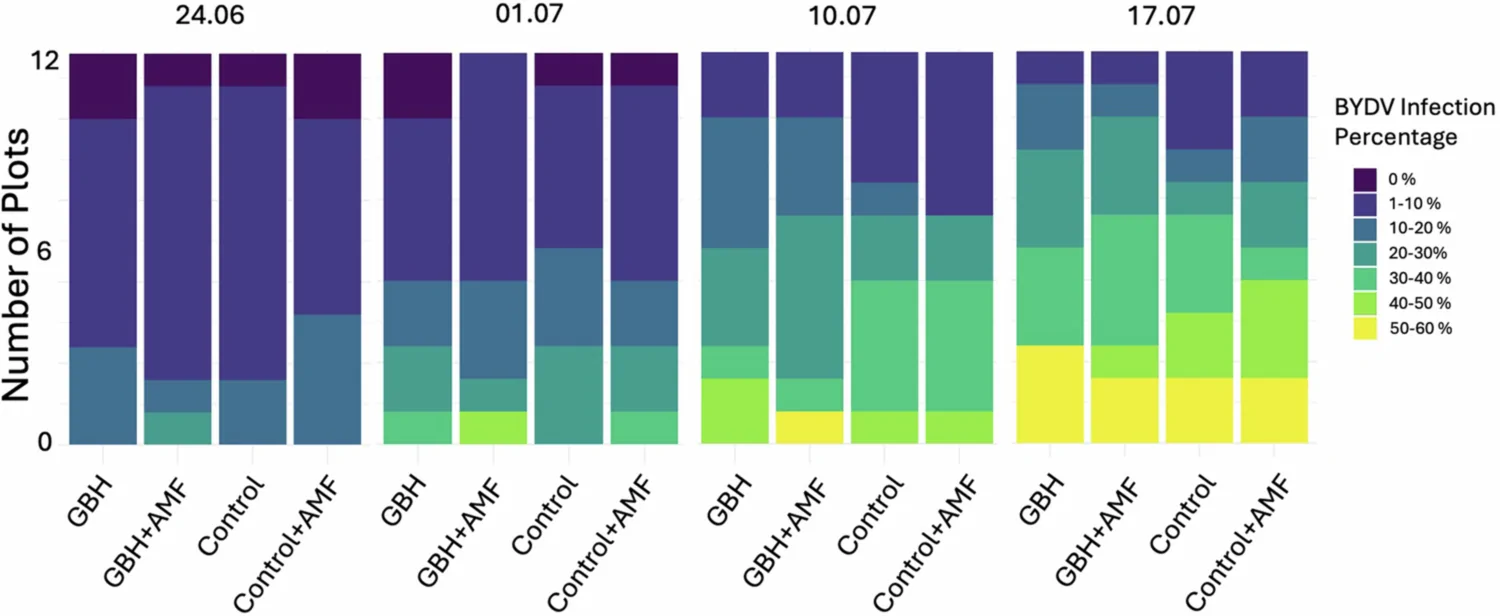
Stacked bar plot showing the distribution of Barley Yellow Dwarf Virus (BYDV) infection percentages across four treatment groups (GBH, GBH + AMF, Control, Control + AMF) at four sampling dates (24.06, 01.07, 10.07, 17.07). Bars represent the number of plots (*n* = 12 per treatment) categorized by infection percentage classes. Color shading indicates infection severity, ranging from 1% to 10% (dark purple) to >60% (bright yellow). A clear increase in infection levels is observed over time. No differences were observed between the treatments per week.

BYDV infection showed visual symptoms on oat plants during the end of June in all treatment groups, which increased during July (Figure 1). At neither time point of assessment did soil treatment, GBH, or AMF treatments impact the degree of virus infection (Figure 1, *p* = 0.7941, week 24.06; *p* = 0.9733, week 01.07; *p* = 0.2683, week 10.07; *p* = 0.915, week 17.07).

### VOC emission

**Figure 2.**
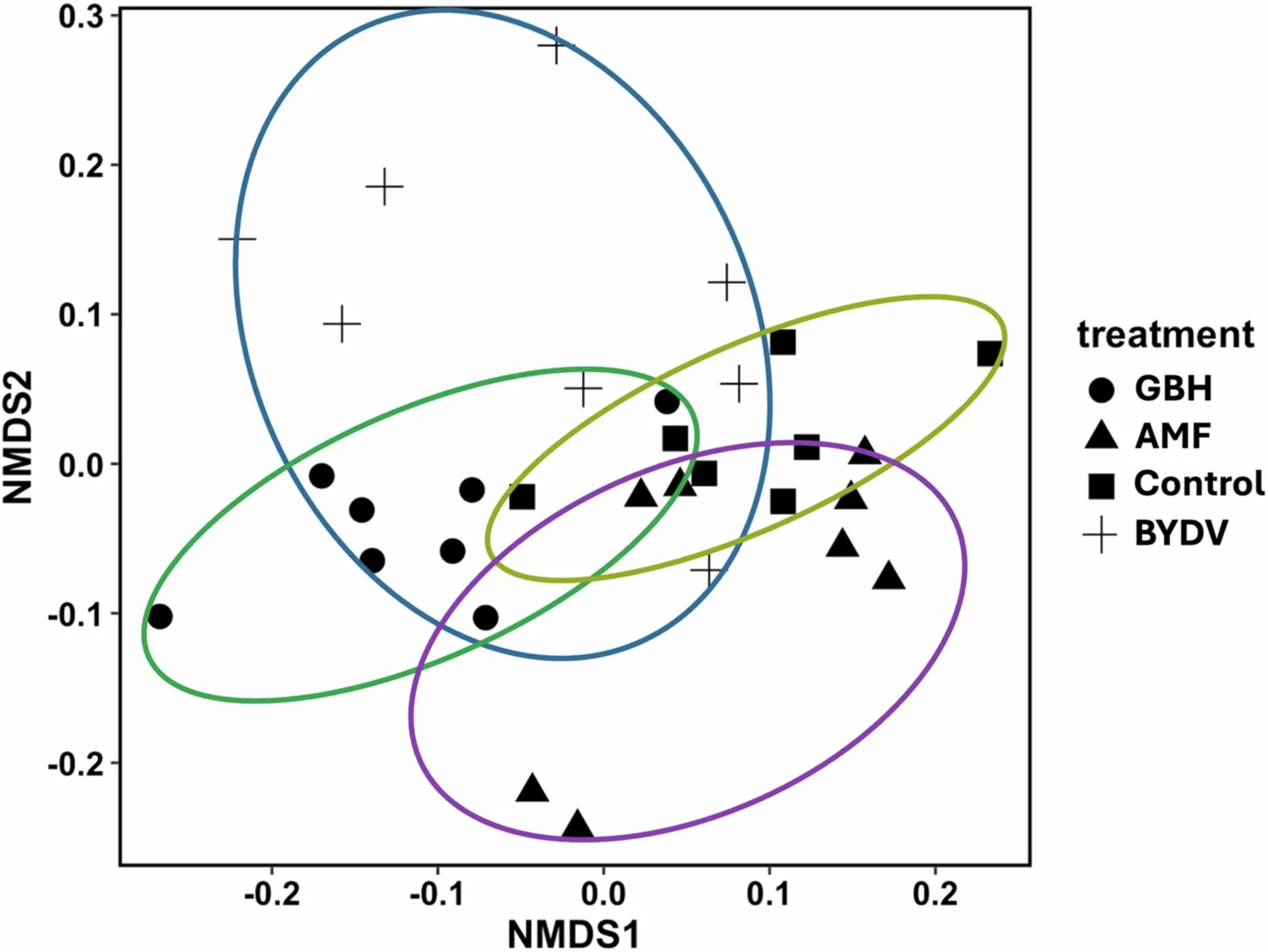
Non-metric multidimensional scaling (NMDS) ordination based on Bray–Curtis dissimilarity of 28 volatile organic compounds (VOCs) across four treatment groups. Each point represents a replicate, and ellipses indicate 95% confidence intervals around group centroids. The ordination reveals treatment-specific clustering of VOC profiles, suggesting compositional differences among treatments. Differences among treatments were tested using PERMANOVA (R^2^ = 0.33, *p* = 0.001).

We observed distinct clustering by treatment group in NMDS space (Figure 2). PERMANOVA based on Bray–Curtis dissimilarity revealed a significant effect of treatment on community composition (R^2^ = 0.33, *p* = 0.001), explained by characteristic VOC patterns (Table 1).

**Figure 3.**
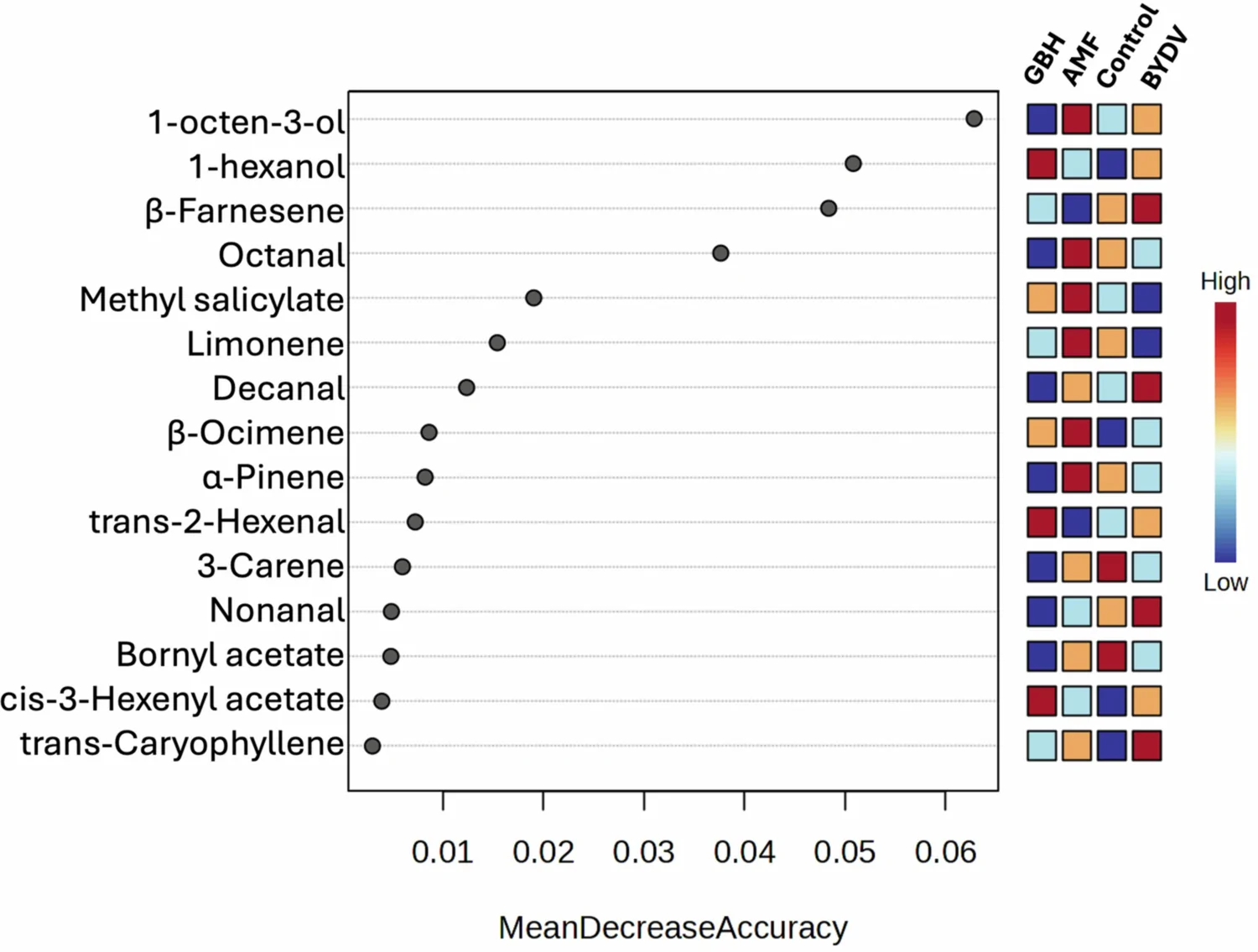
Random Forest analysis of volatile organic compounds (VOCs) emitted under different treatment conditions. Compounds are ranked by their importance for sample classification, measured as the mean decrease in accuracy (left). The accompanying heatmap shows the relative abundance (z-score) of each VOC across the four treatment groups (GBH, AMF, Control, BYDV), with red indicating higher and blue indicating lower relative emissions.

Random Forest analysis revealed distinct volatile emission profiles among the experimental treatment groups, with several compounds contributing disproportionately to their discrimination (Figure 3). Variable importance, measured as the mean decrease in prediction accuracy (MeanDecreaseAccuracy), identified 1-octen-3-ol as the strongest discriminator, exhibiting the highest importance score (Figure 3, 0.063). This compound was followed by 1-hexanol, *β*-farnesene, and octanal, indicating that these metabolites contributed most to the model's ability to correctly classify samples according to treatment. Compounds with intermediate importance included methyl salicylate, limonene, and decanal, whereas *β*-ocimene, *α*-pinene, trans-2-hexenal, 3-carene, nonanal, bornyl acetate, cis-3-hexenyl acetate, and trans-caryophyllene exhibited comparatively lower importance scores, suggesting a more limited contribution to treatment discrimination (Figure 3).

At the individual VOC level, AMF treatment caused an increase in emission of the terpenoid (*E*)-*β*-ocimene, and the fatty acid derivatives 1-octen-3-ol and octanal when compared to the control treatment (Table 1). BYDV infection reduced the emission of octanal and induced the emission of (*E*)-caryophyllene and *β*-farnesene (Table 1). GBH treatment caused an increase in the green leaf volatiles (*Z*)-3-hexen-1-ol, 1-hexanol and (*Z*)-3-hexenyl-acetate compared to controls (Table 1). We only observed individual aphids on infected plants, and the VOC profile was not altered by any herbivore-induced effects.

**Table 1.** Concentrations of individual volatile organic compounds (VOCs) across four treatment groups. Values are presented as mean ± standard deviation (*n* = 8). Different superscript letters within a row indicate statistically significant differences among treatments based on Tukey's HSD post-hoc test (*p* < 0.05). Treatments that do not share a letter are significantly different.

	Emission rate (ng g−1 min−1)			
Compound	Control	AMF	GBH	BYDV
(*E*)-2-Hexenal	0.90 ± 0.26	0.63 ± 0.22	2.54 ± 0.70	1.38 ± 1.04
**(** ***Z*** **)-3-Hexen-1-ol**	**2.93 ** **± 0.81^a^**	**3.79 ** **± 1.10^a^**	**8.80 ** **± 1.48^b^**	**5.64 ** **± 0.93^ab^**
**1-Hexanol**	**1.78 ± 0.31^a^**	**1.88 ** **± 0.39^a^**	**4.67 ± 0.69^b^**	**2.14 ** **± 0.63^a^**
*α*-Pinene	5.49 ± 0.83	5.57 ± 1.38	2.13 ± 0.69	3.75 ± 2.10
Benzaldehyde	0.80 ± 0.15	0.95 ± 0.18	0.48 ± 0.08	0.79 ± 0.33
**1-octen-3-ol**	**3.52 ± 0.65^a^**	**11.07 ** **± 1.38^b^**	**1.52 ± 0.55^a^**	**4.23 ** **± 1.14^a^**
*β*-Pinene	0.67 ± 0.19	0.56 ± 0.18	0.27 ± 0.12	0.59 ± 0.33
Myrcene	0.69 ± 0.16	0.83 ± 0.26	0.61 ± 0.25	1.18 ± 0.39
**(** ***Z*** **)-3-hexenyl-acetae**	**3.54 ± 1.41^a^**	**4.62 ** **± 1.66^a^**	**11.60 ± 1.30^b^**	**8.15 ** **± 1.97^ab^**
3-Carene	3.24 ± 0.50	2.96 ± 0.66	1.17 ± 0.40	2.59 ± 1.33
**Octanal**	**29.05 **±** 6.28^a^**	**40.61 ** **± 4.08^a^**	**9.51 ± 2.27^b^**	**10.42 ** **± 2.32^b^**
**Limonene**	**5.00 ** **± 0.98^ab^**	**8.35 ** **± 0.99^b^**	**3.48 ± 1.15^a^**	**3.08 ** **± 1.09^a^**
**(** ***E*** **)-*β*-Ocimene**	**2.54 ** **± 0.55^a^**	**7.00 ** **± 0.82^b^**	**4.25 ** **± 0.79^ab^**	**3.36 ** **± 0.69^a^**
1-Octanol	0.39 ± 0.10	0.43 ± 0.12	0.16 ± 0.04	0.33 ± 0.18
Acetophenone	43.80 ± 10.76	38.81 ± 4.87	41.03 ± 5.46	46.76 ± 11.80
Nonanal	50.13 ± 10.33	48.51 ± 9.29	29.09 ± 7.57	58.49 ± 7.68
Terpinen-4-ol	5.11 ± 1.52	5.75 ± 1.15	3.95 ± 0.68	4.44 ± 0.79
**Methyl-Salicylate**	**11.73 ** **± 4.38^a^**	**24.72 ** **± 5.07^b^**	**19.38 ** **± 3.19^ab^**	**5.83 ** **± 1.52^a^**
**Decanal**	**8.62 ** **± 1.60^a^ ^b^**	**9.32 ** **± 2.11^ab^**	**4.18 ** **± 0.98^a^**	**12.87 ** **± 2.35^b^**
Nonanoic acid	5.87 ± 1.10	5.78 ± 0.81	5.52 ± 0.76	5.05 ± 1.21
Bornyl acetate	0.83 ± 0.13	0.79 ± 0.19	0.40 ± 0.06	0.62 ± 0.39
Decanoic acid	5.36 ± 1.36	7.04 ± 2.01	6.92 ± 1.01	4.05 v 1.00
**(** ***E*** **)-Caryophyllene**	**1.81 ** **± 0.27^a^**	**2.58 ** **± 0.28^b^**	**1.87 ** **± 0.21^a^**	**3.16 ** **± 0.43^b^**
*α*-Humulene	0.30 ±0.10	0.39 ± 0.12	0.27 ± 0.09	0.23 ± 0.10
Germacrene	0.31 ± 0.05	0.34 ± 0.04	0.32 ± 0.04	0.32 ± 0.06
***β*-Farnesene**	**0.17 ** **± 0.03^a^**	**0.14 ** **± 0.03^a^**	**0.14 ** **± 0.03^a^**	**0.56 ** **± 0.09^b^**
Pentadecane	4.26 ± 0.64	3.85 ± 0.90	3.25 ± 0.46	3.75 ± 1.05
Caryophyllene oxide	1.63 ± 0.37	1.97 ± 0.61	1.04 ± 0.33	1.20 ± 0.47

## Discussion

The results demonstrate that glyphosate-based herbicides (GBH) treatment of the soil, arbuscular mycorrhizal fungal (AMF) seed inoculation, and barley yellow dwarf virus (BYDV) infection change the volatile organic compound (VOC) emission of oat plants in a treatment-specific pattern. BYDV infection rate was not affected by GBH soil treatment or AMF seed treatment. Concentrations of specific VOCs marked the changes in the emission profiles for each of the treatments, demonstrating that biotic and abiotic factors alter plant emitted VOC profiles. Provided that future studies find consistency across environments and years, these changes may serve as indicators of plant and soil health status and may be utilized in future precision diagnostics technologies. Random Forest analysis indicates that treatment-specific differences in the volatile blend were driven primarily by a relatively small subset of compounds rather than by uniform changes across all detected volatiles. The ranking of variable importance suggests that alcohols (1-octen-3-ol and 1-hexanol), together with selected terpenoids and aldehydes such as *β*-farnesene and octanal, accounted for much of the discriminatory power of the volatile profiles.

Plants respond to environmental stimuli with physiological changes and particular biotic interactions can affect plant biochemical processes.^51-53^ Studies have shown that changes in VOC emission connected to disease infection can be identified prior to detectible visual symptoms. This has resulted in the exploration and development of biosensors able to detect changes in VOC emission as a novel tool for plant disease diagnosis.^34^ ^,^ ^35^ Specific patterns of green leaf volatile emission, for example, have been linked to late blight disease in early stages of infection.^36^ Our results demonstrate that BYDV infection caused an increase in the emission of the sesquiterpenes (*E*)-caryophyllene and *β*-farnesene, simultaneously with a reduced emission of octanal in the tested oat plants. (*E*)-caryophyllene is a sesquiterpene often released from damaged plant tissue.^54^ ^,^ ^55^ In virus-infected plants, changes in (*E*)-caryophyllene emission can alter the behavior of insect vectors, which may include the attraction of aphids or whitefly.^56^ ^,^ ^57^ Some plant viruses manipulate VOC emission by changing the emission of (*E*)-caryophyllene to promote their own transmission.^58^ Furthermore, through altered *β*-farnesene levels, as a result of virus infection, various plant species have been linked to reduced aphid colonization or enhanced dispersal, thereby increasing virus spread and its transmission.^59^ ^,^ ^60^ BYDV has previously been shown to impact these compounds in wheat and barley, indicating that the disease causes common physiological changes in cereal species and that the identified VOC pattern may be a reliable indicator for the infection.^37^ ^,^ ^38^ ^,^ ^61^ In the case of plant health diagnostics for disease control and subsequent treatments and measures to control the development of pests, early detection of infection signs is key to minimize damage and yield loss.^6^ Current precision agriculture technologies utilize primarily optical sensorics for disease diagnostics, such as perceiving changes in chlorophyl level, reactive oxygen species, antioxidant, leaf morphology and cell deformation.^62-64^

Mycorrhiza-plant symbiosis is increasingly acknowledged to benefit a multitude of plant species in regard to resistance and resilience when facing abiotic and biotic stress factors.^65-67^ The health status of agricultural soils are often characterized by their abundance of mycorrhizal taxa where intensive agricultural methods including pesticides and tillage reduces AMF abundance.^21^ ^,^ ^68-70^ Currently, categorizing the state of AMF symbiosis requires rhizosphere sampling and advanced analyzes. Utilizing plant VOC pattern to indicate AMF symbiosis may be an elegant tool in future AMF assessments. Several studies have demonstrated that AMF can modulate plant VOC emissions involved in indirect defense. *Funneliformis mosseae* colonization of common bean (*Phaseolus vulgaris*) increased the emission of (*E*)-*β*-caryophyllene, methyl salicylate, and (*E*)-*β*-ocimene, enhancing attraction of predatory mites.^31^ Similarly, herbivore-attacked tomato plants associated with *F. mosseae* released elevated levels of linalool, *α*- and *β*-pinene, and (*E*)-*β*-caryophyllene.^71^ In wheat (*Triticum aestivum*), colonization by *Rhizophagus irregularis* increased emission of (*E*)-*β*-caryophyllene and (*E*)-*β*-farnesene.^32^ In our study (*E*)-*β*-ocimene, (*E*)-caryophyllene and methyl-salicylate, which are common VOCs involved in attraction of predatory insects,^72^ were induced. Being able to categorize plants AMF status by VOC analyzes may be a useful tool to assess the soil health status and the need for microbial amendments.

Agricultural soils are often polluted with GBH residues and knowledge about its various effects on crop performance and plant insect and plant-microbe interactions are accumulating.^14^ ^,^ ^25^ ^,^ ^26^ ^,^ ^28^ ^,^ ^73^ ^,^ ^74^ Changes in the emission of green leaf volatiles (GLVs) have been observed earlier and may indicate that glyphosate residues in soil affect the lipid composition in plant cell walls as they are the precursors to GLVs.^26^ Earlier studies have demonstrated that the airborne pollutant ozone shifts VOC emission in various plants with cascading effects on insect behavior.^50^ ^,^ ^75^ Consequences of glyphosate-mediated changes in ubiquitous plant volatiles may cause similar mismatches in plant-insect and plant-plant interactions, both of which are increasingly recognized as important for the functioning of ecosystem services.^14^ ^,^ ^76^ Furthermore, soil pollutant-altered microbial compositions in the rhizosphere may shape the plant emitted VOCs.^68^ ^,^ ^77^

At the individual metabolite level, we identified mainly GLVs affected by GBH soil treatment which indicates the impact on cell wall composition and enzymatic cell wall degradation.^78^ GLVs are an enzymatic product of cell wall lipids and often produced by cell damage and cell wall degradation.^79^ While we demonstrate an impact of GBH residues in soil on GLV emission, the exact mechanisms are still to be elucidated.

## Conclusions

Our results show that BYDV infection, AMF seed inoculation, and GBH soil treatment each alter oat VOC emissions in distinct ways, although neither GBH treatment nor AMF treatment affected BYDV infection rates. A relatively small set of compounds, particularly GLVs, alcohols, terpenoids, and aldehydes, drove these treatment-specific volatile signatures. These findings highlight the potential of VOC profiling as a non-invasive tool for assessing plant health, disease status, mycorrhizal associations, and soil legacy effects. Future studies should validate these VOC markers under field conditions across multiple years and investigate the mechanisms linking soil management, microbial interactions, and plant VOC emissions.

## Data Availability

All data supporting this study will be made available upon request.
